# Supplementary material for: Addressing Current Challenges in OSL Dosimetry Using MgB4O7:Ce,Li: State of the Art, Limitations and Avenues of Research
Source: Materials (Basel). 2023 Apr 12;16(8):3051. doi: 10.3390/ma16083051 (PMC10142933; doi:10.3390/ma16083051)
Supplement: Supplementary file 1 [file materials-16-03051-s001.zip › materials-2283087-supplementary.pdf]

Addressing Current Challenges in OSL Dosimetry Using MgB_4O_7:Ce,Li: State of the Art, Limitations and Avenues of Research

Lily Bossin [1,*], Igor Plokhikh [1,2], Jeppe Brage Christensen [1], Dariusz Jakub Gawryluk [2], Yuuki Kitagawa [3], Paul Leblans [4], Setsuhisa Tanabe [5], Dirk Vandenbroucke [4] and Eduardo Gardenali Yukihara [1]

1 Department of Radiation Safety and Security, Paul Scherrer Institute, Forschungsstrasse 111, 5232 Villigen, Switzerland; eduardo.yukihara@psi.ch (E.G.Y.)
2 Laboratory for Multiscale Materials Experiments, Paul Scherrer Institute, Forschungsstrasse 111, 5232 Villigen, Switzerland
3 National Institute of Advanced Industrial Science and Technology (AIST), Ikeda, Osaka 563-8577, Japan
4 Radiology Division, Agfa NV, 2640 Mortsel, Belgium
5 Graduate School of Human and Environmental Studies, Kyoto University, Kyoto 606-8501, Japan
* Correspondence: lily.bossin@psi.ch; Tel.: +41-56-310-54-30

1 OSL and TL intensity - comparison with BeO

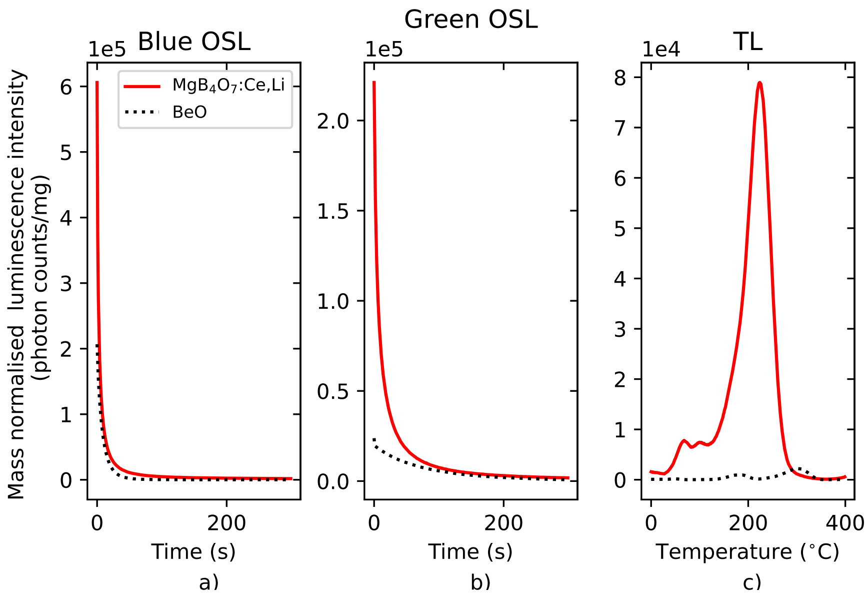

Figure S1:Intensity comparison of the mass normalised signals of MgB_4O_7:Ce,Li and BeO under continuous-wave blue stimulation (a), green stimulation (b) and thermal stimulation (c) following β irradiation 350 mGy. Detection unit: PMT 9235QB; a,b) U-340 filter, c) BG-39 .

*E-mail: lily.bossin@psi.ch

2 Time-resolved luminescence - comparison with BeO

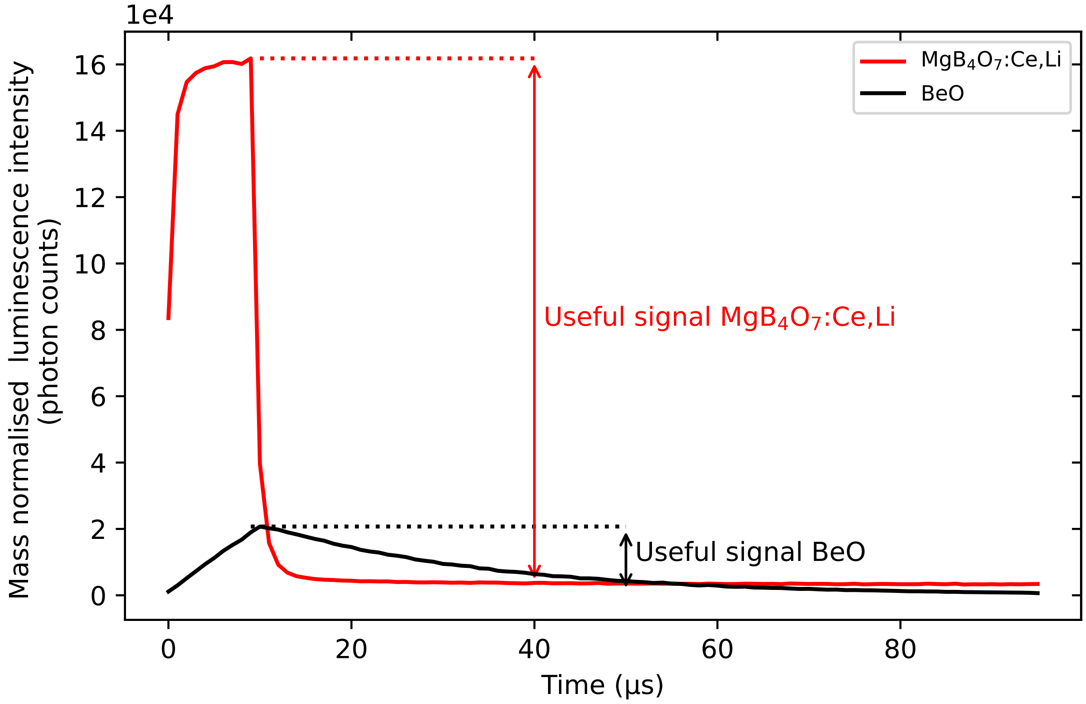

Figure S2: Photon arrival time distribution curves of MgB_4O_7:Ce,Li and BeO following blue stimulation pulse 10 µs, following β irradiation 350 mGy. Detection unit: PMT 9235QB; U-340 filter.
